# Overexpression of P4HA1 associates with poor prognosis and promotes cell proliferation and metastasis of lung adenocarcinoma

**DOI:** 10.7150/jca.63147

**Published:** 2021-09-21

**Authors:** Yue Ning, Hongmei Zheng, Yuting Zhan, Sile Liu, Yang Yang, Hongjing Zang, Qiuyuan Wen, Yajie Zhang, Songqing Fan

**Affiliations:** 1 Department of Pathology, The Second Xiangya Hospital, Central South University, Changsha, Hunan, 410011, China.; 2 Department of Pathology, School of Basic Medical Science, Guangzhou Medical University, Guangzhou, Guangdong 511436, P.R. China.

**Keywords:** P4HA1, non-small cell lung cancer, proliferation, metastasis, biomarker

## Abstract

Prolyl 4-hydroxylase subunit alpha 1 (P4HA1) is the core active catalytic portion of prolyl 4-hydroxylase, and has contributed to tumorigenesis in several cancers. In this study, we identified that P4HA1 mRNA and protein are both up-regulated in non-small cell lung cancer (NSCLC). Besides, overexpressed P4HA1 is correlated with poor clinical outcomes and serve as an independent prognosis biomarker in lung adenocarcinoma (LUAD), but not lung squamous cell carcinoma (LUSC). *In vitro* studies, decreased P4HA1 significantly inhibits proliferation and cell cycle, by regulating cyclin-dependent kinases (CDKs), cyclins and CDK inhibitor (CKI). Moreover, via inhibiting epithelial-mesenchymal transition (EMT) and matrix metalloprotease (MMPs), dysregulation of P4HA1 could restrain the tumor cell invasion and metastasis of lung adenocarcinoma. In addition, we found that P4HA1 could enhance cell stemness and cisplatin-resistance in lung adenocarcinoma. In summary, P4HA1 plays a crucial role in the development of NSCLC and may provide a brand-new target for lung cancer treatment.

## Introduction

Lung cancer, as a type of prevailing malignant tumor, led to considerable mortality and morbidity worldwide 1. Over 85% of lung cancer is non-small cell lung cancer (NSCLC), in which lung squamous cell carcinoma (LUSC) and lung adenocarcinoma (LUAD) are two major categories 2. Common therapies for early-stage NSCLC include surgical resection, chemotherapy and radiation therapy, but they all have undesirable side effects 3. Lately, personalized precision therapy has received much clinical attention with less adverse effects, that explicitly targets tumor cells according to the gene mutation of patients 4. However, patients with NSCLC have a poor prognosis with 15.9% of 5-year survival rate 2. Hence, it is important to find better prognosis biomarkers of NSCLC.

The prolyl 4-hydroxylase subunit alpha 1 (P4HA1) is a member of the prolyl 4-hydroxylase (P4Hs), which catalyzes the post-translational modification and modulates protein folding and stability 5, 6. P4H is an α_2_β_2_ tetrameric, and the α subunit owns peptide binding and catalytic activity. In mammalian there have found three P4HA subtypes including of P4HA1/2/3. P4HA1 being the major isoform of P4H exists many human tissues, and contributes a lot to the prolyl 4-hydroxylase activity 5, 6. It has previously been observed that P4HA1 implicated in the tumorigenesis of various cancers. In prostate cancer, P4HA1 promotes tumor invasion and metastasis via decreasing the expression of tumor suppressor FLRT3 and increasing the expression of oncogenes such as MMPs 7. P4HA1 also could increase the occurrence of tumor proliferation, invasion, metastasis and chemoresistance in mammary cancers 8-10. Overexpressed P4HA1 relates closely tumor cell proliferation and angiogenesis in glioma. Recent studies about pancreatic ductal adenocarcinoma revealed that P4HA1-HIF-1α as a crucial regulator involved in glycolysis and oncogenic activities, such as proliferation, chemoresistance, and stemness 11. However, there has been no detailed investigation of the role of P4HA1 in lung cancer. In our study, we identified that P4HA1 increased in both lung adenocarcinoma and lung squamous cell carcinoma. Furthermore, P4HA1 was correlated with poor prognosis and served as an independent prognosis biomarker for lung adenocarcinoma, but not lung squamous cell carcinoma. Regarding mechanism, we found that P4HA1 regulated EMT and MMPs and further blocked cell cycle and promoted cell proliferation, invasion and migration. Besides, dysregulation of P4HA1 inhibited the stemness and chemoresistance of lung adenocarcinoma. Consequently, P4HA1 may be a promising therapeutic target for lung adenocarcinoma.

## Material and Methods

### Patients and samples

The Cancer Genome Atlas (TCGA) - The Genotype-Tissue Expression (GTEx) Lung Cancer data were downloaded from the University of California Santa Cruz Xena Browser (https://xenabrowser.net) 12-14. Deleting the recurrence cancer samples, a total of 1359 lung cancer and normal cancer samples (513 primary lung adenocarcinoma and 59 solid tissue normal, 498 primary LUSC samples and 51 solid tissue normal, 289 normal lung tissue) remained. Gene Expression Omnibus (https://www.ncbi.nlm.nih.gov/geo/) 15 lung cancer samples were downloaded for the meta-analysis about the P4HA1 expression and prognostic value.

### Ethical statement

All protocols were approved by The Second Xiangya Hospital of Central South University Ethics Review Board (Scientific and Research Ethics Committee, No. S039/2011) and all research was performed in accordance with relevant guidelines/regulations. All research samples were obtained with written informed consent. If the patient is juvenile, a written consent will be signed by caretakers, or guardian on behalf of the juvenile participating in this study.

### Patient cohorts and tissue microarrays (TMAs)

Between 2002 and 2012, we collected 500 NSCLC patients, which diagnosed with NSCLC and underwent surgery and 103 cases of non-cancerous control lung tissues from The Second Xiangya Hospital, Central South University's. All tumors were assessed by expert pathologists using the WHO lung cancer histological classification and the Eighth Edition Lung Cancer to establish tumor stage. At the time of the original procedure, no patients had received radiation or chemotherapy, and none of them had received therapy targeting PD-1/PD-L1 throughout the follow-up period. The time from diagnosis to death, or the final known moment of survival, was used to calculate overall survival time. The Ethics Committee of Central South University's Second Xiangya Hospital accepted this study (No: S039/2011), and all patients with written informed consent had access to comprehensive clinical and follow-up data. In this study, we used the TMA technology to construct high-throughput NSCLC TMAs according to rules previously described 16.

### Exclusion and inclusion criteria

Eligible patients included in this article for TMAs are in accordance with the following inclusion criteria: (1) NSCLC; (2) Complete follow-up data and clinicopathological data. The detailed clinic parameters of enrolled patients were presented in Table S4.

Exclusion criteria included the following: (1) Other treatments were used after the operation; (2) Missing follow-up or clinic parameters.

### Immunohistochemistry and scores

The immunohistochemistry experiment was conducted following the protocol our former study 17. The dilution of primary antibody to P4HA1 was 1:200 (Goat polyclonal antibody, Catalogue GTX89145; GeneTex, North, American). Positive control slides were included in every experiment. The specificity of the antibody was determined with matched IgG isotype antibody as negative control. Expression of P4HA1 was evaluated independently by SF and QW, who were blinded to the clinicopathological data, at 200x magnification light microscopy. Staining intensity for the above markers was scored as 0 (negative), 1 (weak), 2 (moderate) and 3 (strong) as previous described 18. P4HA1 was divided into negative expression and positive expression, which P4HA1 was defined as IHC 1+, 2+ or 3+ regardless of the percentage of positive-stained cells. Agreement between the two evaluators was 95%, and all discrepancies were resolved through discussion. Staining scores ≤2 and >2 was regarded as low/high expression, respectively, for an optimal cut‐off level for P4HA1 protein.

### Cell lines

The non-neoplastic lung epithelial cell 16HBE, BEAS-A2 and six NSCLC cell lines (A549, H460, H1299, H520, H1975 and SPC-A1) were obtained from the ATCC (American type culture collection). Cell lines were cultured in RPMI-1640 medium (Thermo Fisher Scientific, USA) containing 10% fetal bovine serum (Gibco, USA). The culture dishes were placed in the incubator at 37 °C, 5% CO_2_.

### Lentivirus infection

The lentiviral vectors were designed and synthesized by landbiology (Guangzhou, China). The target sequence of shRNA is as follows: P4HA1: 5′-CTAGTACAGCGACAAAAGA-3′. The infection process was performed according to the manufacturer's instructions. A549 and SPC-A1 cells were infected with a multiplicity of infection (MOI) of 2 and incubated for 8 hours. Three days after infection, cells were cultured in 10% FBS medium with 2.5 μg/ml puromycin (Sigma-Aldrich).

### qRT-PCR

Total RNA was extracted using Trizol RNA reagent (Themo Fisher Scientific, USA). Reverse transcription was performed using the PrimeScript™ RT reagent Kit with gDNA Eraser (Takara, Japan). qRT-PCR was conducted using SYBR Green™ Premix Ex Taq™ II (Takara, Japan) in Applied Biosystems 7500 (AB, USA). The primers were as follows: GAPDH, forward ACAACTTTGGTATCGTGGAAGG; reverse GCCATCACGCCACAGTTTC; P4HA1 forward GGAACAAGCCCTAAGG CAACT; reverse TGCTGATATACCGCATAGCTCAA.

### Western blot analysis

Western blotting was performed according to the standard protocol 19 with the following antibodies: GAPDH, CDK1/2/4/6, P21 (CST), MMP2, MMP9, ZO-1, E-cadherin, Vimentin, N-cadherin, OCT4, CCNB1, SOX2 (Abcam), P4HA1 (GeneTex and Proteintech), NANOG (Proteintech).

### Cell counting kit-8 (CCK-8) and colony formation assays

Cells were planted into 96-well plates with 1000 cells per well. CCK-8 reagents (Djingo, Japan) were added at every at 24h for five days. The optical density was estimated at 450 nm wavelength. Cells were planted in in 6-well plates (500/well) and incubation for 2 weeks at 37 °C, 5% CO_2_. Colonies were washed triple with PBS and stained with crystal violet for 15min.

### Cell-cycle analysis

Cell for cell cycle analysis were harvested after 48 h incubation and washed with PBS. The cells were further fixed with 70% ice-cold ethanol at 4 °C overnight. The samples were sent to Sun Yat-Sen hospital for further experiments.

### Migration and invasion assays

Migration and invasion assays were performed using Transwell chambers (Corning USA). For migration assays, cells (2×10^5^ cells) were seeded with serum-free medium onto the top chamber, and the bottom chamber was filled with 10% FBS medium. For invasion assays, BD Matrigel were first added in the top chambers for 2h, and then cells (2×10^5^ /well) were planted. After 18h, all the chambers collected and fixated with methanol for 30 min, stained with 0.01% crystal violet for 15 min.

### Stem Cell Sphere Formation Assay

Cells (1×10^3^ cells/well) were plated in 24-well plates with ultra-low adherence (Corning, USA) and cultured in DMEM/F12 medium, supplemented with B27, 20 ng/ml EGF and 20 ng/ml bFGF for 14 days to form spheres.

### Gene Set enrichment analysis (GESA)

Genes co-expression with P4HA1 were extracted using Pearson's correlation analysis (|r| ≥ 0.3) in cBioPortal (http://www.cbioportal.org/) 20, 21 with TCGA lung adenocarcinoma cohort. Gene Ontology (GO) and Kyoto Encyclopedia of Genes (KEGG) pathways analysis were conducted in DAVID website (https://david.ncifcrf.gov/summary.jsp) 22.

### Statistical analysis

GraphPad Prism version 8.0 (GraphPad Software, Inc.) or R version 3.6.2 were used for data analysis. Unless specifically mentioned, all values are presented as the mean ± SD. Two groups had equal variances, two‑group independent sample comparisons Student's t‑test (two‑tailed) were performed, while Welch's t‑test was used for unequal variances. Multi‑group samples statistics were analyzed via one‑way ANOVA if the variances were equal; if not, Welch's ANOVA was performed. Bonferroni post hoc tests were performed for all ANOVAs. Samples from TCGA Lung Cancer Cohort were divided into high and low expression groups according to median values. DSS, PFI, OS, DFI curves were plotted using the Kaplan‑Meier method, log‑rank test was used to assessed differences. Cox proportional hazard regression model was hired to estimate the prognostic role of P4HA1 in the patients with NSCLC. *P*<0.05 was considered to indicate a statistically significant difference.

## Results

### P4HA1 is highly expressed and exists prognosis value in non-small cell lung cancer

**Figure 1:**
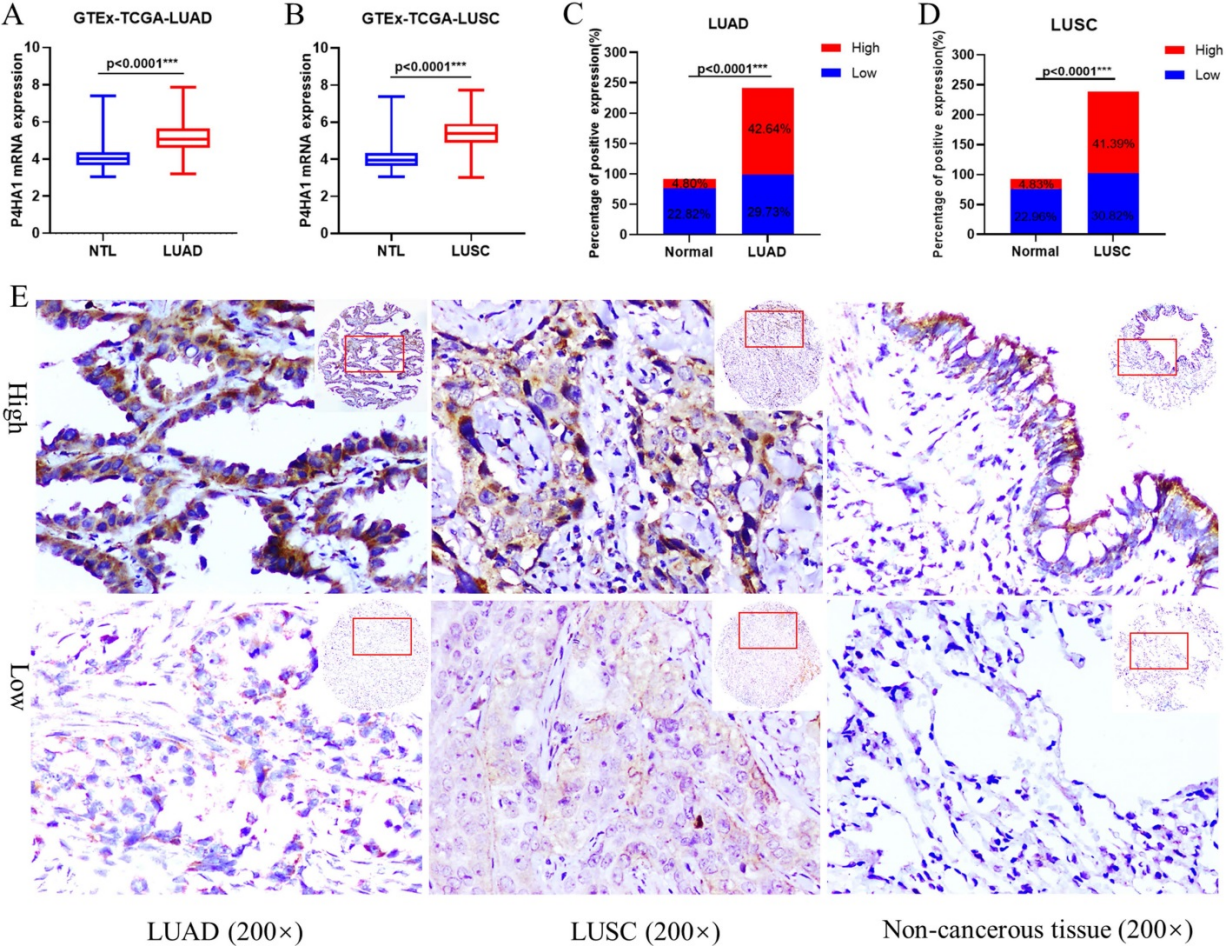
** P4HA1 is upregulated in lung cancer. (A-B)** P4HA1 mRNA expression in lung adenocarcinoma and lung squamous cell carcinoma compared with normal lung tissues based on samples from GTEx-TCGA database. **(C-D)** P4HA1 protein expression in lung adenocarcinoma and lung squamous cell carcinoma compared with non-cancerous lung tissues based on tissue microarray. **(E)** Representative images of immunohistochemistry show differences in P4HA1 expression between lung adenocarcinoma, lung squamous cell carcinoma and non-cancerous lung tissues. ^*^P<0.05 was considered to indicate a statistically significant difference. ^***^ stands for P<0.0001.

GTEx-TCGA lung cancer cohort having the largest samples were first selected to assessed the mRNA expression of P4HA1 in our study. In the cohort, P4HA1 showed distinct differential expression in both lung adenocarcinoma and lung squamous cell carcinoma compared with normal lung tissue (Figure 1A-B). Besides, we conducted a meta-analysis of P4HA1 mRNA expression in 31 NSCLC GEO cohorts from different countries and sources. According to the results, we found that P4HA1 mRNA highly expressed in most NSCLC cohort (24/31) compared with those in non-tumor tissues, except GSE11969, GSE39345, GSE31552, GSE43767, GSE63459, GSE19804 (Table S1). Nevertheless, expression of P4HA1 in blood sample showed no difference between NSCLC patients and healthy person (GSE20189). We further verified the expression level of P4HA1 mRNA in the NSCLC and adjacent normal lung tissues. The results showed that P4HA1 was highly expressed in 16/20 patients (Figure S1A).

Next, we explored the P4HA1 protein expression in our NSCLC tissue arrays by Immunohistochemistry. We found that P4HA1 protein increased in lung cancer tissues compared to non-cancerous lung tissues in both lung adenocarcinoma (Figure 1C) and lung squamous cell carcinoma (Figure 1D). P4HA1 located in the cytoplasm and membrane of cancer cells, and rarely identified in nucleus (Figure 1E). Western blot analysis on fresh lung tissue samples revealed that P4HA1 protein were high expressed in 16/20 samples (Figure S1B).

**Figure 2:**
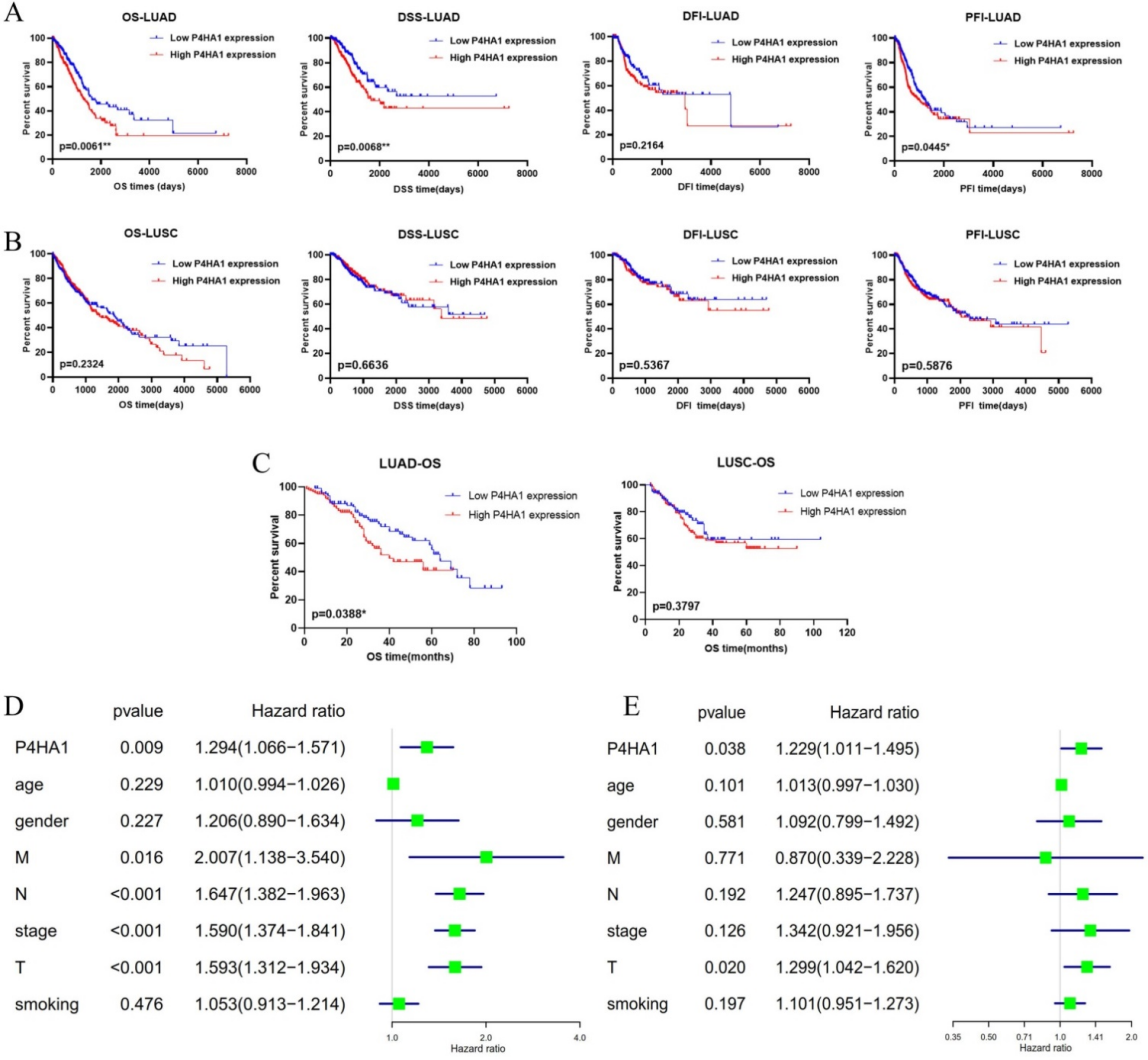
** High P4HA1 expression confers poor prognosis for lung adenocarcinoma. (A-B)** Kaplan-Meier OS, DSS, DFI and PFI survival analysis of lung adenocarcinoma and lung squamous cell carcinoma patients from TCGA database based on P4HA1 mRNA expression. **(C)** Kaplan-Meier overall survival analysis of lung adenocarcinoma and lung squamous cell carcinoma patients based on P4HA1 protein expression. **(D-E)** The Univariate and Multivariate analysis of OS and P4HA1 mRNA expression and clinical-pathological features in TCGA lung adenocarcinoma patients.

We also analyzed with TCGA lung samples with follow-up data that the prognostic value of increased expression of P4HA1 in lung adenocarcinoma and lung squamous cancer, separately. Kaplan-Meier analysis revealed that P4HA1 was negatively correlated to overall survival (OS), disease-specific survival (DSS), and progression free interval (PFI), but not with disease-free interval (DFI) in lung adenocarcinoma (Figure 2A). The results of analysis also showed that P4HA1 expression had no correlation with prognosis in lung squamous cancer (Figure 2B). Then we rushed GEO datasets with intact clinical information to validate the prognostic value of P4HA1 (Table S2). Further detection for the effect of P4HA1 protein expression on OS of LUAD and LUSC indicated that elevated P4HA1 protein was related to poor overall survival of LUAD, but no significant relation was detected in P4HA1 protein expression with OS of LUSC (Figure 2C).

Moreover, we investigated with the Cox regression, both univariate (Figure 2D) and multivariate (Figure 2E) process to validate whether P4HA1 could serve as an independent prognostic biomarker in lung adenocarcinoma. The results of Table 1 present that P4HA1 can be a potential predictor for lung adenocarcinoma OS, rather than DSS and PFI (Table S3).

Taken together, these results indicated that P4HA1 might serve as an independent prognostic biomarker for lung adenocarcinoma patients. Since P4HA1 displayed greater prognostic value in lung adenocarcinoma, we will focus on lung adenocarcinoma in our further study.

### Correlation of P4HA1 expression with clinicopathological features in lung adenocarcinoma

To investigate the role of P4HA1 expression in lung adenocarcinoma progression, we assessed the association of clinicopathological features with P4HA1 mRNA and protein expression (Table 1). Our analysis in the TCGA‑LUAD Cohort demonstrated that P4HA1 mRNA expression was related with tumor diameter and distant metastasis. Also, we found no significant relation in P4HA1 mRNA expression with age, gender, lymph node metastasis and stage. Concerning the relations in P4HA1 protein expression and clinicopathological features, we found P4HA1 protein expression presented association with only tumor diameter, which consistent with the mRNA results. Hence, we hypothesized that P4HA1 may promote proliferation and distant metastasis of lung adenocarcinoma.

### Identification of biological processes and signaling pathways P4HA1 involved

To get insight into the biological functions and potential molecular pathways of P4HA1 implicated in the tumorigenesis of lung adenocarcinoma, we carried on the Gene enrichment analysis with TCGA LUAD Cohort. Firstly, we analyzed the co-expression genes of P4HA1 in cBioPortal. Genes with |r| ≥ 0.3 were further used to Gene ontology analysis and KEGG pathways analysis in DAVID. GO analysis revealed that P4HA1 involved in multiple biological processes, including cell proliferation, hypoxia response and cell cycle (Figure S2A). KEGG pathways analysis results indicated that P4HA1 implicated in cell cycle, metabolism, pathway in cancer and HIF-1 signaling pathways (Figure S2B). In summary, all of the analysis above suggests that P4HA1 may be a valuable modulator in lung adenocarcinoma.

### Decreased P4HA1 inhibits proliferation of lung adenocarcinoma cells

**Figure 3:**
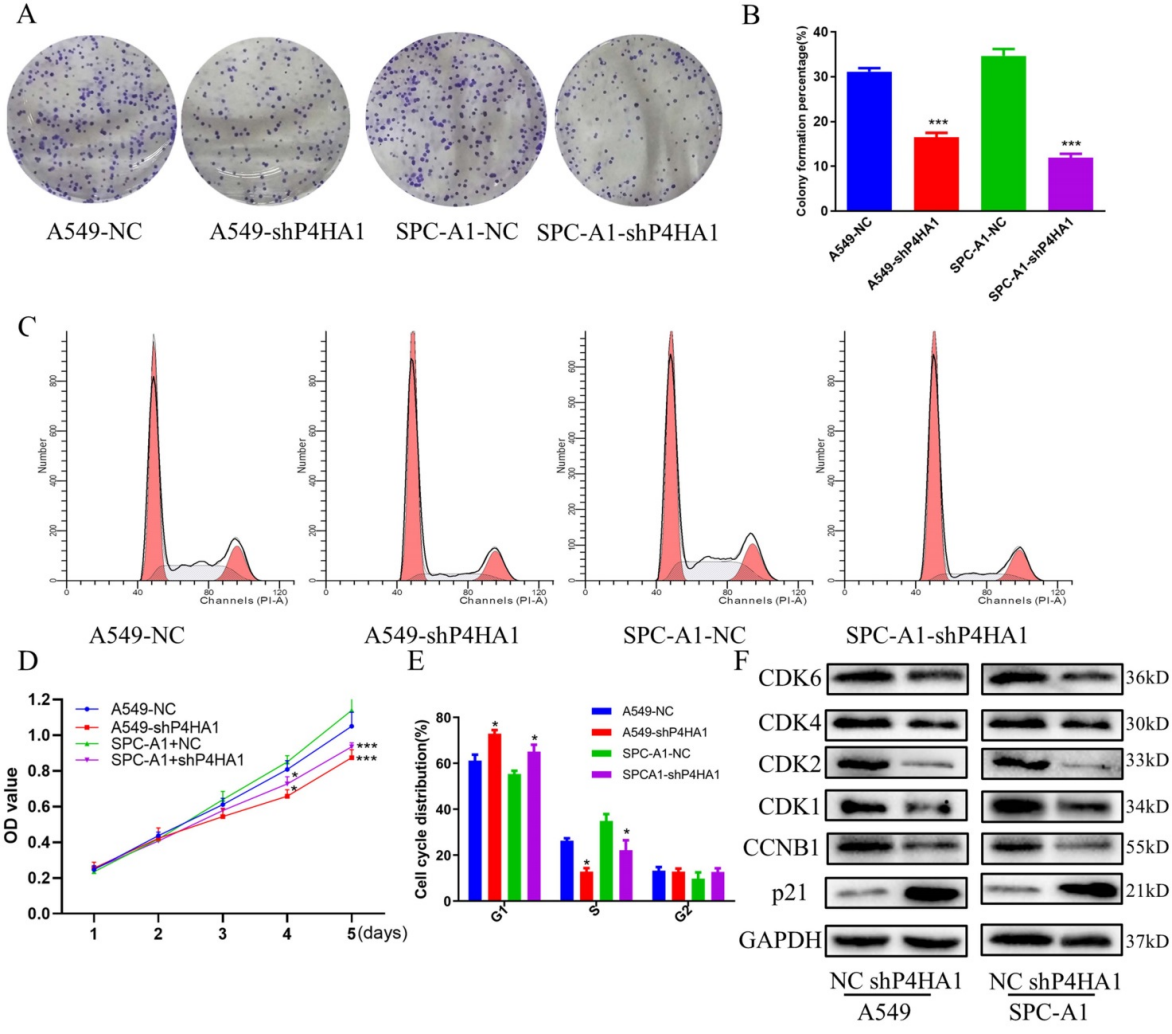
** P4HA1promotes proliferation of lung adenocarcinomas. (A, D)** Colony formation assays, (**B**) CCK-8 assays and (**C, E**) flow cytometry analyses of P4HA1-konckdown A549 and SPC-A1 cells and in the corresponding control groups. (**F**). Western blot analysis was used to examine proliferation signaling-associated protein expression levels in lung adenocarcinoma cells.

We investigated P4HA1 expression in six different lung cancer cells and human bronchial epithelial cell line with real-time PCR and Western blotting. The results indicated P4HA1 was highly expressed in six lung cancer lines in both mRNA and protein level, in which A549 and SPC-A1 were the two most high expressed adenocarcinoma cell lines (Figure S1C-D). qPCR and western blotting results demonstrated that P4HA1 shRNA evidently decreased the expression of P4HA1 (Figure S1E-F), which NC stands for control group, while shP4HA1 stands for P4HA1 shRNA knockdown group. Colony formation proved that knockdown P4HA1 significantly blocked proliferation of A549 and SPC-A1 cells (Figure 3A-B, D). We used flow cytometry cell cycle to identify whether knockdown P4HA1 could inhibit proliferation of lung adenocarcinoma cells via affecting cell cycle. The results indicated that compared to control group, the fraction in G1 phase increased while S phase decreased in P4HA1 cells (Figure 3C, E). Thus, knockdown of P4HA1 induced cell cycle arrest in the G1 phase. The western blot results indicated knockdown P4HA1 inhibited cyclin-dependent kinases (CDKs) CDK1/2/4/6 and cyclin proteins CCNB1, while increased expression of CDK inhibitor (CKI) P21 (Figure 3F).

### P4HA1 promotes cell migration and invasion of lung adenocarcinoma

**Figure 4:**
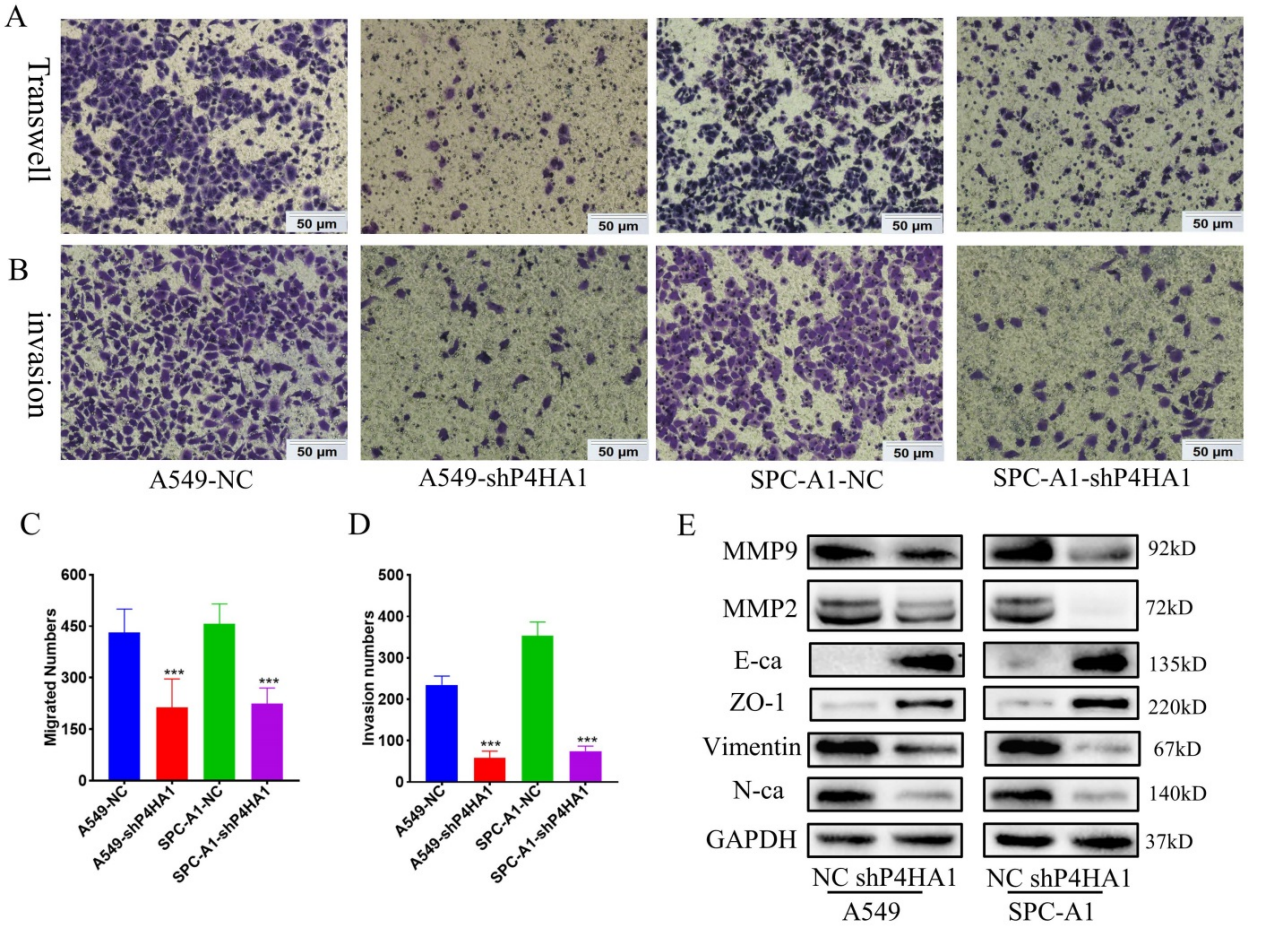
** P4HA1promotes migration and invasion of lung adenocarcinomas. (A, C)** Transwell assays and (**B, D**) invasion assays of P4HA1-konckdown A549 and SPC-A1 cells and corresponding control groups (**E**). Western blot analysis was utilized to examine metastasis signaling-associated protein expression levels in lung adenocarcinoma cells.

Transwell migration assays demonstrated that knockdown P4HA1 inhibited the ability of tumor cell migration in lung adenocarcinoma. Moreover, Matrigel invasion assay indicated that knockdown of P4HA1 significantly inhibited tumor cell invasion of lung adenocarcinoma (Figure 4A-D). In terms of the mechanism, P4HA1 knockdown showed features of epithelial-mesenchymal transition (EMT), that decreased N-cadherin, Vimentin, Snail and increased E-cadherin and ZO-1 (Figure 4E). In addition, knockdown of P4HA1 inhibited the expression of MMP2 and MMP9, which were widely considered to relate to cancer invasion.

### Knockdown P4HA1 lessens the stem cell-like phenotype in lung adenocarcinoma

**Figure 5:**
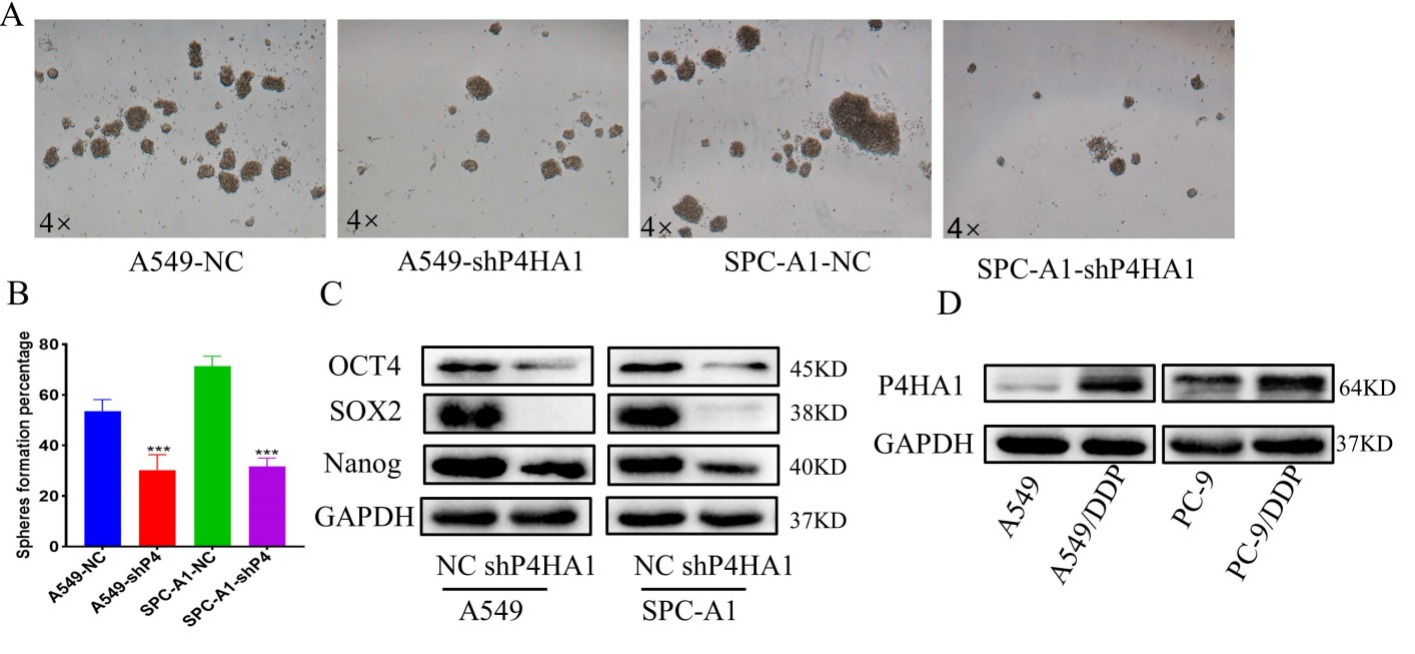
** P4HA1 promotes stemness and cisplatin of lung adenocarcinoma. (A-B)** Stem sphere formation assays of P4HA1-konckdown A549 and SPC-A1 cells and their corresponding control groups. (**C**). Western blot analysis was utilized to examine cancer stemness-related protein expression levels in lung adenocarcinoma cells. **(D)** P4HA1 expression levels in CDDP resistant cells and parental cells.

To detect the function of P4HA1 in the stemness of lung adenocarcinoma, we applied cancer cell sphere formation analysis. From the results, we found that knockdown P4HA1 formed smaller and fewer stem spheres compared to the corresponding groups (Figure 5A-B). Besides, lung adenocarcinoma cells with P4HA1 knockdown showed decreasing OCT4, SOX2, NANOG protein levels (Figure 5C).

### Inhibition of P4HA1 sensitizes lung adenocarcinoma to chemotherapy

Previous studies have shown that inhibiting P4HA1 could make triple-negative breast cancer sensitive to chemotherapy. We detected P4HA1 expression in cisplatin-resistant cell lines to demonstrate whether P4HA1 is associated with chemoresistance in lung adenocarcinoma. The expression of P4HA1 in cisplatin-resistant lung adenocarcinoma remarkably increased compared with control groups (Figure 5D).

## Discussion

P4HA1 is the key component of the rate-limiting enzyme P4H, which plays a vital role in synthesis of various collagens 6, 23. Previous studies from many hypoxia-related microarray analyses have identified that P4HA1 exists significant changes in response to hypoxia. However, only several researches have stated the role of P4HA1 in tumor diseases, and most of them are breast cancer related 24-27. In present study, we have identified P4HA1 function as an oncogene in NSCLC through the combination of bioinformatic analyses, molecular biology, and cell biological experiments.

Many studies have reported that P4HA1 overexpresses and serves as a satisfying prognostic biomarker. In mammary cancer, P4HA1 showed a dramatical high-expression on both mRNA and protein level, and P4HA1 mRNA expression was correlated with shorter overall survival times 9, 10. In glioma, P4HA1 protein was found up-regulated, and downregulation of P4HA1 associated with extended OS of xenograft mice 28. In prostate cancer and pancreatic cancer, P4HA1 increased both on mRNA and protein levels, and P4HA1 expression was correlated with poor overall survival in pancreatic ductal adenocarcinoma patients 7, 11. In our study, we identified that P4HA1 highly expressed both in LUAD and LUSC samples. Besides, overexpressed P4HA1 mRNA was negatively correlated with OS, DSS, PFI, but not correlated with DFI in LUAD or LUSC in TCGA samples. We further validated our results in GEO lung cancer datasets. And we demonstrated that P4HA1 protein expression was correlated with poor overall survival in lung adenocarcinoma, and was not correlated in lung squamous cell carcinoma. Furthermore, univariate and multivariate regression analysis confirmed P4HA1 mRNA acted as an independent prognostic factor for OS, but not DSS and PFI in LUAD.

After analyzing the relationship between P4HA1 and clinical pathological parameters of lung adenocarcinoma patients, we found P4HA1 mRNA expression relating to tumor diameters and distant metastasis, while not relating to age, gender, smoking history and clinical stage. However, P4HA1 protein expression was associated with stage, which was different from mRNA level. Additionally, Gene Ontology and KEGG pathways enrichment analyses suggested that P4HA1 could regulate proliferation and metabolism. Previous studies reported that P4HA1 promoted proliferation, metastasis in breast cancer, prostate cancer, and glioma 7, 9, 10, 28. Therefore, our hypothesis was that P4HA1 may involve in the proliferation and metastasis of lung adenocarcinoma. Our *in vitro* study validated that decreased P4HA1 expression inhibited the proliferation, migration and invasion of lung adenocarcinoma cells. According to the gene enrichment analysis, P4HA1 may regulate the proliferation of lung adenocarcinoma by regulating cell cycle. Flow cytometry cell cycle analysis further proved that knockdown P4HA1 inhibited G1/S transition of cell cycle. MMPs and EMT are the most common mechanism for cancer metastasis. In prostate cancer, P4HA1 could promote prostate cancer metastasis via regulating MMP1 expression 7. In ovarian cancer cells, miR-122 suppressed EMT by targeting P4HA1 29. Similarly, our results indicated that P4HA1 promoted tumor metastasis through regulating the MMPs and EMT. Newly reports have verified that P4HA1 plays an essential role in the process of glioma stem cells (GSCs) into endothelial cells (ECs) trans differentiation 28. Hence, we assumed that P4HA1 might involve in stem cell function of lung adenocarcinoma. In this study, our cancer cell sphere formation analyses firstly demonstrated that knockdown P4HA1 increased the numbers and diameter of lung adenocarcinoma cell spheres. Chemotherapy applies widely in treatments to patients with lung cancer. Early study had shown that inhibiting P4HA1 increased chemosensitivity in triple-negative breast cancer 28. Consistently, our study demonstrated that P4HA1 remarkably increased in displacing resistant cell lines.

Our study firstly proves the functional of P4HA1 in the progression of NSCLC disease. These results suggest that P4HA1 may act as a potential drug target and prognostic indicator for lung adenocarcinoma. Consequently, the development of highly specific and potent P4HA1 inhibitors may be the key to overcome chemotherapy resistance and improve lung cancer treatment.

## Supplementary Material

Supplementary figures and tables.

## Figures and Tables

**Table 1:** Correlation of P4HA1 expression level with the clinicopathological features in lung adenocarcinoma

P4HA1 mRNA				P4HA1 protein				
Variable	Number	P4HA1	P value	Variable	Number	Low	High	P
**Age (years)**								
<65	149	10.99±0.7374		≥ 55	132	52 (24.19%)	80 (37.21%)	
≥65	194	10.93±0.7530	0.4622	< 55	83	32 (14.88%)	51 (23.72%)	0.9022
**Gender**								
Female	175	10.93±0.7011	0.5859	Female	93	37 (17.21%)	56 (26.05%)	0.8511
Male	168	10.98±0.7911		Male	122	47 (21.86%)	75 (34.88%)	
**T**								
T1 and 2	103	10.74±0.7044	0.0005^***^	T1 and 2	172	73 (33.95%)	99 (46.05%)	0.0427^*^
T3 and 4	240	11.05±0.7457		T3 and 4	43	11 (5.12%)	32 (14.88%)	
**LNM status**								
LNM	218	10.93±0.7431	0.5277	N0	86	34 (15.81%)	52 (24.19%)	0.9091
No LNM	125	10.99±0.7522		N1-3	129	50 (23.26%)	79 (36.74%)	
**M**				**Pathological degree**				
M0	317	10.93±0.7371	0.0432^*^	Well/moderated	131	53 (24.65%)	78 (36.28%)	0.6023
M1	26	11.26±0.8189		Poor	84	31 (14.42%)	53 (24.65%)	
**Clinical stages**								
I and II	261	10.93±0.7256	0.2208	I and II	109	49(22.79%)	60(27.91%)	0.0729
III and IV	82	11.04±0.8052		III and Ⅳ	106	35(16.28%)	71(33.02%)	

**Note:** * *P* < 0.05; ** *P* <0.01; *** *P* <0.001.
