# Supplementary material for: Overexpression of P4HA1 associates with poor prognosis and promotes cell proliferation and metastasis of lung adenocarcinoma
Source: J Cancer. 2021 Sep 21;12(22):6685–94. doi: 10.7150/jca.63147 (PMC8517996; doi:10.7150/jca.63147)
Supplement: Supplementary file 1 — Supplementary figures and tables. [file jcav12p6685s1.pdf]

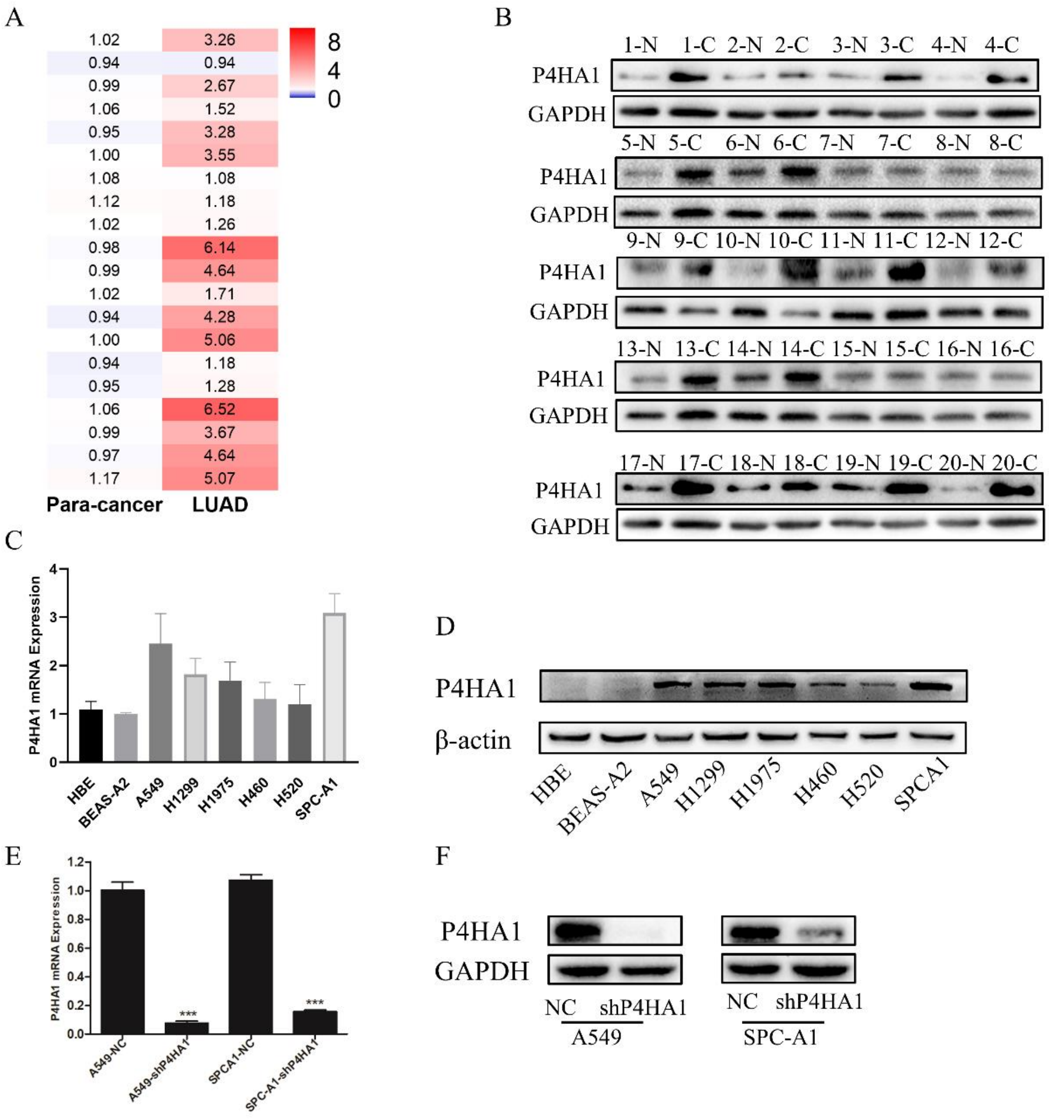

Figure. S1 P4HA1 expression in lung cancer tissue and lung cancer cell lines. (A-B). The mRNA and protein expression level of P4HA1 in 20 paired samples from patients with lung cancer (primary cancer and para-cancer tissues). (C-D). P4HA1 mRNA and protein expression in six lung cancer cell lines. (E-F). shRNA knockdown P4HA1 in mRNA and protein levels.

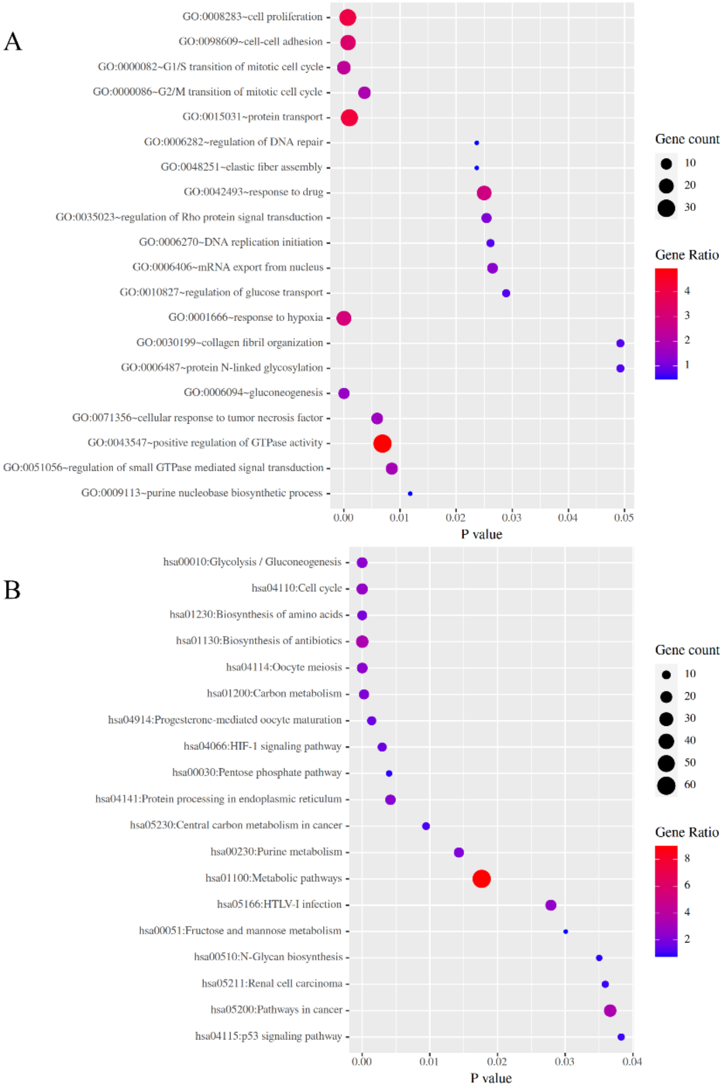

Figure. S2 The function of P4HA1 in lung adenocarcinoma. Gene Enrichment Analysis based KEGG pathways (A) and Gene Ontology (B) utilized co-expression genes.

Table S1. Expression of P4HA1 in TCGA and GEO NSCLC datasets

Author	Source	Country	Series	Cancer		Normal		*P* value
				N	Mean ± SD	N	Mean ± SD	
NSCLC								
Baty F	Tissue	Switzerland	GSE11117	15	9.11±0.78	41	10.33±0.10	0.0100*
Takeuchi T	Tissue	Japan	GSE11969	5	0.11±0.20	158	0.22±0.15	0.1165
Ramos AS	Tissue	Spain	GSE18842	45	9.02±0.28	46	10.10±0.76	<0.0001***
Philipsen S	Tissue	Netherlands	GSE19188	65	-0.43±0.47	91	0.31±0.82	<0.0001***
Li-Jen S	Tissue	Taiwan	GSE27262	25	0.88±0.16	25	1.46±0.83	0.0013**
Sophie	Tissue	France	GSE30219	14	8.68±0.25	211	9.37±0.79	0.0012**
Meister M	Tissue	Germany	GSE33532	20	7.92±0.29	80	9.29±0.65	<0.0001***
Chen Y	Tissue	Taiwan	GSE39345	20	4.53±1.36	32	3.93±1.64	0.2122
Kastner S	Tissue	Austria	GSE40275	43	6.94±0.36	41	7.88±0.80	<0.0001***
Seo J	Tissue	USA	GSE40419	77	20.83±3.50	87	30.70±15.00	<0.0001***
Khadijah	Tissue	USA	GSE101929	34	10.75±0.35	32	11.49±0.52	<0.0001***
Rotunno	Blood	USA	GSE20189	81	6.71±0.40	49	6.70±0.43	0.9469
Marquardt G	Tissue	USA	GSE31552	68	7.41±1.70	63	7.99±1.69	0.0545
Arima C	Tissue	Japan	GSE51852	4	0.38±0.13	49	1.40±0.79	0.0130*
LUAD								
Su L	Tissue	Taiwan	GSE7670	27	9.72±0.71	27	10.58±0.99	0.0010**
MariaTeresa	Tissue	USA	GSE10072	49	9.40±0.29	58	10.08±0.54	<0.0001***
David Q	Tissue	USA	GSE32665	92	10.20±0.67	87	11.03±0.59	<0.0001
Suhaida A	Tissue	USA	GSE32863	58	6.96±0.07	58	7.02±0.12	0.0026**
Kadara H	Tissue	USA	GSE43458	30	8.74±0.35	80	9.12±0.68	0.0037**
Feng L	Tissue	China	GSE43767	15	8.44±0.41	69	8.84±0.79	0.0611
Robles AI	Tissue	USA	GSE63459	32	6.95±0.10	33	7.00±0.12	0.0602
Mervi	Tissue	USA	GSE68571	10	8.53±0.64	86	9.31±0.86	0.0296**
Gazdar A	Tissue	USA	GSE75037	83	4.05±0.30	83	4.32±0.60	0.0003**
Bossé Y	Tissue	Canada	GSE83213	46	9.16±0.19	11	9.59±0.50	<0.0001***
Rezzonico R	Tissue	France	GSE116959	11	10.13±0.22	57	10.68±0.66	0.0078**
Mervi	Tissue	USA	GSE68645	19	11.33±0.10	443	10.55±0.67	<0.0001***
TCGA-LUAD	Tissue	TCGA	LUAD	59	10.24±0.25	514	10.98±0.78	<0.0001***
Tzu-Pin	Tissue	Taiwan	GSE19804	60	3.54±0.13	60	3.57±0.14	0.1271
Kohno T	Tissue	Japan	GSE31210	20	11.08±0.83	226	11.74±0.11	<0.0001***
LUSC								
Mascaux C	Tissue	USA	GSE33479	27	1.11±0.34	27	1.53±0.98	0.0419*
TCGA-LUSC	Tissue	TCGA	LUSC	502	10.25±0.28	51	11.20±0.83	<0.0001***

Note: NSCLC: non-small cell lung cancer; LUAD, lung adenocarcinoma; LUSC, squamous lung carcinoma; $^{*}P<0.05$; $^{**}P<0.01$; $^{***}P<0.001$.

Table.S2 Meta-analysis prognostic value of P4HA1 in GEO

GEO datasets	Author	Country	OS			FPS		
			sample counts	HR	Log-rank p value	sample counts	HR	Log-rank *P* value
LUAD								
GSE30219	Sophie	France	85	4.26(1.51-11.98)	0.2335			
GSE3141	Joseph	USA	54	3.2(1.4-7.29)	0.0037**			
GSE31210	Kohno T	Japan	226	3.32(1.38-8.01)	0.0045**	226	2.39(1.37-4.17)	0.0015**
GSE50081	Ming Tsao	Canada	125	1.51(0.86-2.66)	0.1481	124	1.52(0.79-2.92)	0.2015
GSE37745	Patrick	Sweden	106	1.38(0.87-2.19)	0.1705			
GSE68465	Mervi	USA	443	1.291(0.9981-1.670)	0.0483*	178	1.142(0.8446-1.546)	0.3875
GSE68571	Mervi	USA	86	1.151(0.5135-2.578)	0.9678			
GSE8894	Jhinkook Kim	Korea				63	2.37(1.15-4.91)	0.017*
LUSC								
GSE14814			52	0.43(0.15-1.2)	0.0982			
GSE30219	Sophie	France	61	0.65(0.31-1.37)	0.2528			
GSE3141	Joseph	USA	53	1.39(0.63-3.07)	0.4093			
GSE4573	Jack Yu	USA	130	1.63(0.98-2.72)	0.0592			
GSE37745	Patrick	Sweden	66	1.68(0.97-2.92)	0.0636			
GSE8894	Jhinkook Kim	Korea				75	2.09(1.05-4.19)	0.033*

Note: LUAD, lung adenocarcinoma; LUSC, lung squamous cell carcinoma; OS, overall survival; FPS, free progression survival; * $P<0.05$; ** $P<0.01$.

Table.S3 The univariate and multivariate analysis of OS, PFI, DFI, DSS of P4HA1 and clinical-pathological in TCGA

Gene	PFI - Univariate Cox		PFI - Multivariate Cox		DSS - Univariate Cox		DSS - Multivariate Cox	
	HR	p-value	HR	p-value	HR	p-value	HR	p-value
**Age**	0.994(0.977-1.011)	0.481	0.996(0.978-1.013)	0.625	0.980(0.958-1.003)	0.092	0.993(0.970-1.016)	0.540
**Gender**	1.016(0.730-1.414)	0.926	0.963(0.682-1.359)	0.829	1.017(0.641-1.611)	0.944	0.883(0.552-1.414)	0.605
**Smoking**	1.167(0.996-1.367)	0.056	1.199(1.019-1.412)	0.030*	1.138(0.921-1.408)	0.232	1.205(0.960-1.857)	0.097
**T**	1.416(1.147-1.748)	0.001**	1.230(0.961-1.576)	0.101*	1.626(1.211-2.183)	0.001*	1.335(0.960-1.857)	0.086
**N**	0.605(1.082-1.634)	0.007*	0.970(0.680-1.382)	0.865	1.594(1.224-2.075)	<0.001*	1.273(0.804-2.016)	0.304
**M**	1.558(0.838-2.895)	0.161	0.672(0.240-1.881)	0.450	2.468(1.226-4.966)	0.016*	1.395(0.347-5.602)	0.639
**Stage**	1.330(1.14-1.583)	0.001**	1.324(0.881-1.988)	0.177	1.572(1.270-1.946)	<0.0001***	1.230(0.707-2.139)	0.464
**P4HA1**	1.437(0.927-2.227)	0.105	1.079(0.865-1.345)	0.500	1.226(0.917-1.638)	0.052	1.171(0.869-1.579)	0.301

Note: * P<0.05; ** P <0.01; *** P <0.001.

Table S4. Clinicopathological characteristics of patients with non-small cell lung cancer (NSCLC) and non-cancerous lung tissues in the tissue microarrays

Patient characteristics	No. of patients (%)
**NSCLC**	
**Age (years)**	
<55	178 (39.2)
≥55	276 (60.8)
**Gender**	
Male	343 (75.5)
Female	111 (24.5)
**T**	
T1-2	317 (69.8)
T3-4	137 (30.2)
**Clinical stages**	
Stage I and II	244 (53.7)
Stage III	210 (46.3)
**Lymph node status**	
No LNM	230 (50.7)
LNM	224 (49.3)
**Histological type**	
SCC	239 (52.6)
ADC	215 (47.4)
**Differentiation**	
Well and moderate	269 (59.2)
Poor	185 (40.7)
**Survival status**	
Alive	329 (72.5)
Dead	125 (27.5)
**non-cancerous lung tissues**	
**Age(years)**	
<55	47 (51.1)
≥55	45 (48.9)
**Gender**	
Male	63 (68.5)
Female	29 (31.5)
